# Kommunalfinanzierung in der Corona-Krise — Einschnitte, aber keine Zeitenwende

**DOI:** 10.1007/s10273-021-2824-6

**Published:** 2021-01-19

**Authors:** Stephan Brand, Johannes Steinbrecher

**Affiliations:** KfW Research, Abtl. Volkswirtschaft, KfW Bankengruppe, Palmengartenstr. 5-9, 60325 Frankfurt am Main, Deutschland

**Keywords:** H60, H74, H81

## Abstract

Über die vergangenen Jahre stellte sich der Status quo der Kommunalfinanzierung in Deutschland als recht stabil dar. Der Kommunalkredit ist dabei insbesondere für die Investitionen eine zentrale Säule der Finanzierung. Größere Veränderungen waren in den letzten Jahren sowohl auf der Nachfrage- wie auf der Angebotsseite nicht zu beobachten. Die wirtschaftlichen Folgen der Corona-Pandemie könnten für beide Marktseiten nun eine ungewohnte Dynamik mit sich bringen. Positiv ist in diesem Zusammenhang hervorzuheben, dass sich die Schuldentragfähigkeit der Kommunen im Vorkrisenzeitraum spürbar verbessert hat. Auch wenn die Folgen der Krise noch nicht abschließend beurteilt werden können, dürfte der Kommunalkredit somit auch in den nächsten Jahren eine wichtige Rolle bei der Finanzierung kommunaler Investitionen spielen.

## Abstract (en)

Over the last few years, the role of municipal loans for financing investment in Germany has been quite stable. Major changes have not been observed on either the demand or the supply side. The economic consequences of the Coronavirus pandemic could now create unexpected stress for both sides of the market. Even if the consequences of the crisis cannot yet be fully assessed, debt sustainability of municipalities has improved in the pre-crisis period creating financial leeway. Thus, municipal loans are likely to continue to play an important role in the financing of municipal investments in the years to come.

Die Kommunen nehmen über ihre Leistungen der öffentlichen Daseinsvorsorge und ihre Investitionstätigkeit eine wichtige Rolle für die Wettbewerbsfähigkeit und Lebensqualität einer Region bzw. des Wirtschaftsstandorts Deutschland insgesamt ein. Kommunalkredite sind dabei ein wichtiger Baustein für die Finanzierung kommunaler Leistungen, insbesondere der Investitionen. Die Kommunalfinanzierung wird allerdings ebenso wie nahezu alle anderen Wirtschaftsbereiche durch die Folgen der Corona-Krise beeinflusst, die sich vielfältig auf die öffentlichen Haushalte auswirken wird (Freier und Geißler, 2020; oder Bender et al., 2020). Sowohl mit Blick auf das Kreditangebot als auch die Kreditnachfrage stellen sich krisenspezifische Herausforderungen, die im Rahmen dieses Beitrags diskutiert werden sollen.

**Dr. Stephan Brand** und **Dr. Johannes Steinbrecher** arbeiten als volkswirtschaftliche Referenten mit Schwerpunkt auf Kommunen und Infrastruktur bei KfW Research in Frankfurt am Main. Sie vertreten hier die eigene Meinung und nicht notwendigerweise die der KfW Bankengruppe.

## Rolle des Kommunalkredits im Finanzierungsmix

Die Kommunalfinanzierung in Deutschland wird auf der Nachfrageseite vor allem von der Investitionstätigkeit der Städte, Gemeinden und Landkreise bestimmt. Neben den anderen kommunalen Einnahmequellen sehen die Gemeinde- und Haushaltsordnungen die Finanzierung über Fremdkapital als subsidiären Weg vor, wenn die sonstigen Mittel nicht ausreichen bzw. deren Einsatz nicht wirtschaftlich wäre (Schwarting, 2019; oder Zimmermann und Döring, 2019). Darum sind Kredite eigentlich nur für (rentierliche) Investitionen zulässig, um die finanzielle Belastung auf der Zeitachse zu verteilen (Brand und Steinbrecher, 2017a).[1]

Ausnahme von dieser sehr restriktiven Finanzierungspolitik stellen die Kassenkredite dar, welche die Kommunen aufnehmen können, um ihre Liquidität bei Schwankungen der Einnahmen und Ausgaben sicherzustellen. Diese „Dispokredite“ sind jedoch zeitnah, d. h. in der Regel unterjährig zu tilgen.[2] Eine hohe, persistente Kassenkreditverschuldung gilt als Alarmsignal für eine angespannte Finanzlage und steht aktuell im Zentrum der Debatte um

1 Die Kommunen müssen ihre dauerhafte, finanzielle Leistungsfähigkeit im Blick behalten, also auch die Kreditkosten aus Zins und Tilgung decken können, sodass eine Überschuldung eigentlich nicht möglich sein sollte. Problematisch ist jedoch die Bestimmung einer Rentabilität öffentlicher Investitionen und damit auch der tragbaren Folgekosten (Zimmermann, 2006).

2 Jedoch sind auch längere Laufzeiten von Kassenkrediten zu beobachten und der permanente Aufwuchs seit den 2000er Jahren ließ den Verdacht zu, dass diese Kredite zunehmend zweckentfremdet als Dauerfinanzierungsinstrument in Kommunen mit Haushaltslöchern genutzt werden (Brand, 2016).

Abbildung 1
**Instrumente der kommunalen Investitionsfinanzierung**

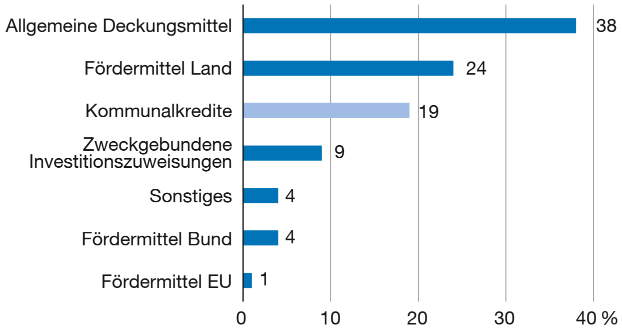

Anmerkungen: Dargestellt ist der Anteil der jeweiligen Finanzierungsmittel am Investitionsvolumen einer Kommune.

Quelle: KfW-Kommunalpanel 2020.

die kommunalen „Altschulden“ (Brand und Steinbrecher, 2018a, 2018b). Demgegenüber ist eine hohe investive Verschuldung nicht zwangsläufig ein Hinweis auf Finanzschwäche, da sich die Investitionstätigkeit – und damit auch der Kreditbedarf – stark an der Haushaltslage orientiert (Brand und Steinbrecher, 2020a oder 2017b).

Bei der Investitionsfinanzierung tendieren die Kommunen zu einem eher konservativen Instrumentenmix (vgl. Abbildung 1). Zumeist werden Investitionen über Eigenmittel und Investitionszuweisungen bzw. Fördermittel gedeckt (Krone und Scheller, 2020). Gerade die Eigenmittel, die sich vor allem aus den Schlüsselzuweisungen des Kommunalen Finanzausgleichs sowie den eigenen Steuereinnahmen zusammensetzen, bestimmen die investiven Haushaltsspielräume und damit auch die Finanzierungsbedarfe bzw. -möglichkeiten. Diese Einnahmearten dürften zugleich am stärksten von der Krise beeinträchtigt werden. Über Kredite finanzieren die Kommunen aktuell rund ein Fünftel des Investitionsvolumens, wobei immerhin 70 % der Kommunen in Deutschland Kredite nutzen.[3]

## Entwicklung der kommunalen Kreditnachfrage

Die Brutto-Kreditaufnahme der Kommunen in den Kern- und Extrahaushalten lag 2019 bei 10,9 Mrd. Euro (Statistisches Bundesamt, 2020a).[4] Sie bewegte sich damit in der Bandbreite von 10 Mrd. bis 12 Mrd. Euro der Vorjahre. In der Summe betrug die Verschuldung 2019 131,4 Mrd. Euro, darunter 95,5 Mrd. Euro Investitions- und 32,9 Mrd. Euro Kassenkredite (Statistisches Bundesamt, 2020b). In den vergangenen Jahren konnte der Schuldenstand insgesamt deutlich reduziert werden, der 2015 mit über 144,2 Mrd. Euro einen vorläufigen Höhepunkt erreicht hatte. Dies ist zum Großteil auf die Reduktion der Kassenkreditbestände zurückzuführen. Dieser Trend dürfte durch die wirtschaftlichen Folgen der Corona-Krise gebrochen werden. Die Verschuldung der Gemeinden und Gemeindeverbände erhöhte sich im 1. Halbjahr 2020 gegenüber dem Jahresende 2019 um 1,1 Mrd. Euro auf jetzt 132,4 Mrd. Euro. Die fiskalischen Folgen der Krise werden in den öffentlichen Haushalten aber erst in den weiteren Folgequartalen vollständig zutage treten, weil sich die wirtschaftlichen Auswirkungen mit Verzögerung in den Kassen zeigen und nochmals später statistisch ausgewiesen werden.[5]

Auffällig bei der Verschuldung ist, dass die hochverschuldeten Kommunen seit 2010 deutlich weniger Erfolge bei der Schuldenrückführung verzeichnen konnten als niedriger verschuldete Kommunen (vgl. Abbildung 2a). Während die Pro-Kopf-Verschuldung beim am niedrigsten verschuldeten Viertel der Kommunen (unterstes Quartil) von 2010 bis 2018 um fast 20 % zurückgegangen ist, hat das am höchsten verschuldete Viertel im gleichen Zeitraum die (nominale) Verschuldung nicht substanziell verringern können. Bereits vor Ausbruch der Corona-Krise haben sich somit spürbare Unterschiede bei der kommunalen Verschuldung gezeigt, die sich nun durch die Krise verschärfen dürften.

## Marktstrukturen für Kommunalkredite

Unter den gemeindlichen Schulden stellt der Kommunalkredit mit über 90 % das gängigste Instrument der Fremdfinanzierung dar.[6] Alternative Finanzierungswege werden nur selten und tendenziell in größeren Kommunen

3 Es handelt sich dabei um die gewichtete Zahl der an der Befragung zum KfW-Kommunalpanel teilnehmenden Kommunen, welche zur Zeit der Befragung Kommunalkredite nutzen, mit mehr als 2.000 Einwohner:innen, die für insgesamt über 90 % aller kommunalen Investitionen verantwortlich sind (Krone und Scheller, 2020).

4 Weil die Kommunen 2019 dank Überschüssen ihre Verbindlichkeiten in Höhe von 10,9 Mrd. Euro tilgen konnten, lag die Netto-Kreditaufnahme gerade mal bei 15 Mio. Euro. Dies deckt sich jedoch nicht mit der Schuldenstatistik, die für das gleiche Jahr eine Kreditaufnahme beim nicht-öffentlichen Bereich von 11,7 Mrd. Euro sowie eine Tilgung von 11,4 Mrd. Euro ausweist. Neben unterschiedlichen Stichtagen und Abgrenzungen dürfte auch die Erfassung kaufmännisch buchender Extrahaushalte/Zweckverbände in der Schuldenstatistik ein Grund für die Abweichung sein.

5 In der vergangenen Finanzkrise war 2010 das Jahr mit dem größten kommunalen Finanzierungsdefizit, obwohl die Krise ihren Ausgang im Jahr 2008 nahm und das Jahr 2009 den stärksten wirtschaftlichen Einbruch hatte.

6 Zu den genutzten Kreditformen und dem kommunalen Kreditmanagement, vgl. Rehm und Tholen (2008) oder Rehm und Matern-Rehm (2010) sowie Albers und Rohloff (2007).

Abbildung 2
**Entwicklung der Pro-Kopf-Verschuldung und des Durchschnittszinses**

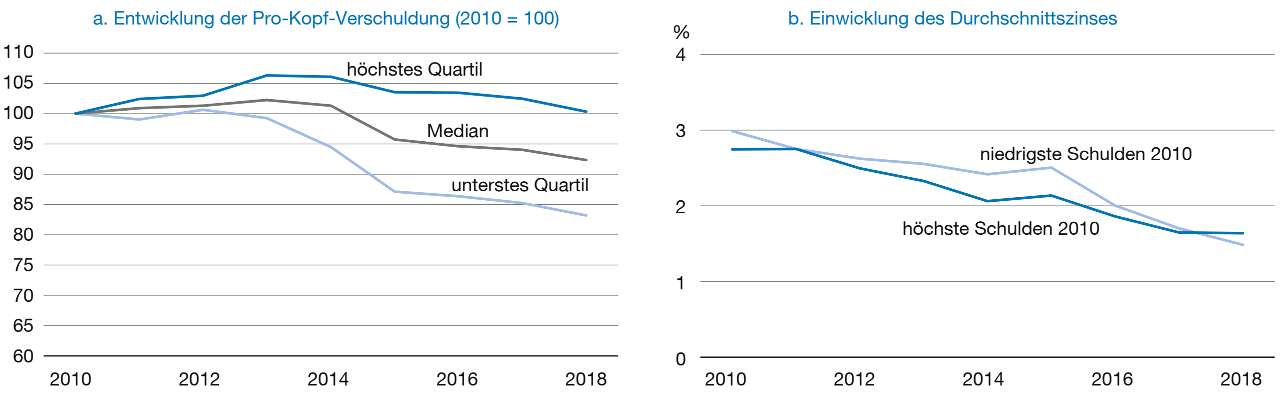

Anmerkungen: Dargestellt ist die Entwicklung der Pro-Kopf-Gesamtverschuldung für alle Gemeinden mit mehr als 2.000 Einwohner:innen anhand des Medians sowie des 25 %- und 75 %-Quartil der Pro-Kopf-Verschuldung (Abbildung 2a). In Abbildung 2b ist die durchschnittliche Schuldenlast anhand des Verhältnisses von Zinsausgaben zu Gesamtverschuldung abgebildet, unterteilt in die Gruppe der höchsten und niedrigsten Verschuldung im Jahr 2010. Die Unterteilung wurde wie in Abbildung 2a anhand von Quartilen vorgenommen.

Quelle: eigene Darstellung nach Kassen- und Schuldenstatistik (v. J.), Statistisches Bundesamt.

gewählt. Hier sind vor allem Schuldscheindarlehen und vereinzelt auch Anleihen zu nennen (vgl. Kasten 1).[7] Ein Grund für die dominante Rolle des Kommunalkredits bei der Fremdfinanzierung ist der gut entwickelte Kreditmarkt in Deutschland, der Kommunen eine schnelle, komfortable und tendenziell günstige Finanzierungsmöglichkeit bietet. Viele Kommunen unterhalten dafür Geschäftsbeziehungen mit einer Vielzahl an Banken.[8]

Zuträglich für die Attraktivität des Kommunalkredits war zudem die Niedrigzinsphase der letzten Jahre.[9] So konnten die Kommunen bei der Aufnahme neuer Schulden bzw. der Umschuldung alter Schulden ihre Kreditfinanzierungskosten spürbar senken. Vor allem kurzlaufende Kredite haben sich vergünstigt und waren von einigen Banken sogar zu Negativzinsen erhältlich, weil eine Darlehensvergabe an die Kommunen noch als attraktiver bewertet wurde als das „Parken“ verfügbarer Liquidität bei der EZB zu einem negativen Einlagesatz (Köstler, 2020). Allerdings zeigt sich trotz dieser komfortablen Ausgangslage eine große Streuung bei den Zinslasten

7 Jedoch ist der Anteil der Wertpapierschulden in der Summe nicht über 3 % hinausgekommen. Ein Grund dafür sind die hohen Volumina, die zur wirtschaftlichen Nutzung dieser Instrumente benötigt werden und von den meisten Kommunen nicht erreicht werden (Brand und Steinbrecher, 2019a).

8 Die Zahl der Finanzierungspartner hängt nicht zuletzt von der Größe der Finanzierungsvolumina und damit der Einwohnergrößenklasse ab (Scheller und Schneider, 2017). Im Kontakt zwischen Kommune und Bank haben zuletzt Vermittlungsplattformen ein größeres Augenmerk erhalten. Auch wenn sowohl auf Anbieter- wie Nachfrageseite noch nicht die kritischen Größenordnungen erreicht werden, könnte die Digitalisierung der Kreditprozesse diese schneller, einfacher und damit günstiger machen (Schiereck und Wittmann, 2018).

9 Die Kommunen haben anteilig weniger von der Niedrigzinsphase profitiert als Bund und Länder, die sich stärker über Kapitalmarkinstrumente (insbesondere Anleihen) finanzieren und hier noch höhere Zinsersparnisse realisieren konnten. Demgegenüber stehen gestiegene Baupreise, die auch aufgrund des durch die Niedrigzinsen angeheizten Baubooms die kommunalen Haushalte stärker treffen, weil vor allem die investiven Baumaßnahmen auf kommunaler Ebene eine gewichtige Rolle spielen (Brand und Steinbrecher, 2020a).

Kasten 1
**Rolle der Kapitalmärkte für die Kommunen**

In den vergangenen Jahren zeigten institutionelle Investoren auf der Suche nach sicheren und lukrativen Anlagemöglichkeiten ein steigendes Interesse an Investments in Kommunen (Handelsblatt, 2017). Im Vordergrund standen ESG-konforme Anlagen, für die Kommunen bzw. deren Infrastruktur prädestiniert schienen. Trotzdem spielt der Kapitalmarkt weiterhin eine untergeordnete Rolle, weil Finanzierungsinstrumente wie Anleihen eigentlich nur für wenige große Kommunen in Frage kommen, selbst wenn es z. B. mit dem Klimaschutz beträchtliche Investmentfelder gäbe (Brand und Steinbrecher, 2019a). Als Folge der Krise bleibt abzuwarten, wie viel Kapital Investor:innen zukünftig noch in Kommunen investieren wollen. Zwei gegensätzliche Szenarien sind denkbar: Bleibt das Kapitalangebot hoch und sind die Kämmereien an Alternativen zum Kommunalkredit interessiert, würden Anleihen und Co. zukünftig eine größere Bedeutung erhalten. Reduziert sich jedoch die Bereitschaft der Investor:innen, Kapital für die Kommunalfinanzierung zur Verfügung zu stellen, bleibt der Kommunalkredit weiterhin zentral für die gemeindliche Fremdkapitalfinanzierung.

zwischen den Kommunen. Denn Zinsersparnisse dürften (mit entsprechenden Zinsänderungsrisiken) vor allem bei den Kassenkrediten zu realisieren sein, sodass nicht alle Kommunen gleichermaßen ihre Kreditkosten optimieren können. Dies spiegelt sich dann auch in sehr unterschiedlichen Belastungsindikatoren wie z.B. der Zins-Einnahme-Quote (Brand und Steinbrecher, 2018b). Positiv ist in diesem Zusammenhang, dass der durchschnittliche Zins, den Kommunen auf ihre Schulden zahlen, jedoch sowohl bei niedrig- als auch bei hochverschuldeten Kommunen in den vergangenen Jahren kontinuierlich gesunken ist (vgl. Abbildung 2b). Die Schuldenlast einer Kommune sank somit in der Regel selbst dann, wenn die Verschuldung nicht reduziert werden konnte.[10]

Das niedrige Zinsniveau entlastet somit zwar die Haushalte, hat jedoch wenig Einfluss auf die Höhe der Investitionen und damit die Kreditaufnahme. Weil vor allem politische Bedarfe bei der Investitionsplanung im Vordergrund stehen, sind betriebswirtschaftliche Aspekte zweitrangig. Kommunale Investitionen rentieren sich also nicht eher oder werden verstärkt in Angriff genommen, nur weil die Zinsen niedrig sind (Brand und Steinbrecher, 2020a). Jedoch begünstigen niedrige Kreditkosten dieses Instrument im Vergleich mit anderen Finanzierungswegen. Aufgrund der zuletzt guten Haushaltslage der letzten Jahre in vielen Kommunen nutzten diese aber stärker Eigenmittel oder konnten auf Rücklagen zurückgreifen.

Die relativ geringe Nachfrage nach Krediten im Verhältnis zum Investitionsvolumen ist somit primär auf die Verfügbarkeit von Alternativen zurückzuführen und nicht auf die Konditionen des Kommunalkredits. Diese wurden in den vergangenen Jahren durchgehend als positiv bewertet. Unter den Kommunen, die Kredite nutzen, bewerten immerhin 42 % der befragten Kämmereien im KfW-Kommunalpanel die Finanzierungsmöglichkeiten inklusive der verfügbaren Konditionen als gut, 48 % sogar als sehr gut (Krone und Scheller, 2020). Nur ein Zehntel bewertet die Möglichkeiten neutral oder negativ. Dies wird vor allem damit begründet, dass die Zahl der Angebote kleiner ausfällt. Eine Verschlechterung der Rahmenbedingungen erwarteten – noch vor der Corona-Krise – nur weniger als 10 % der befragten Kommunen. Die überwiegend positive Einschätzung hat sich auch in der Corona-Krise bislang nicht grundlegend geändert: im Mai 2020 gaben in einer Ergänzungsumfrage immer noch 65 % der befragten Kommunen an, dass sie keine Verschlechterung der Kreditfinanzierungsmöglichkeiten aufgrund der Corona-Krise befürchten (Brand, Steinbrecher und Krone, 2020). Stattdessen planten 36 % eine zusätzliche Aufnahme von Kassenkrediten und 26 % von Investitionskrediten, um coronabedingte Einnahmeausfälle zu kompensieren. Zudem hatten bereits vor der Krise 49 % der Kommunen eine wachsende Bedeutung des Kommunalkredits erwartet, sodass zumindest auf der Nachfrageseite von keinem Einbruch in der Kommunalfinanzierung auszugehen ist, wenngleich Aspekte der Schuldenkonsolidierung für die öffentliche Hand nach der Krise wieder stärker in den Vordergrund rücken dürften.

## Kommunalkredite nach Bankengruppen

Auf der Angebotsseite wird die Kommunalfinanzierung mit den Förderbanken, den Landesbanken und den Sparkassen von drei Bankengruppen besonders stark geprägt und stellte sich in den letzten Jahren als recht stabil dar (Freier und Grass, 2013; oder Brand, 2015). Einen größeren Umbruch gab es ab 2004, als im Zuge der sogenannten „Brüsseler Konkordanz" 2001 die Landesbanken aufgespalten und die Infrastrukturfinanzierung auf die nun eigenständigen Landesförderinstitute übertragen wurden (vgl. Abbildung 3a). Die Landesbanken konzentrierten sich auf ihr eigenes Landesgebiet oder auf andere Geschäftsbereiche, sodass ihr Anteil an der Kommunalfinanzierung abnahm (Osman, 2008; oder Seikel, 2013). Die Förderbanken hingegen konnten ihre Bedeutung in der Kommunalfinanzierung steigern (Keck, 2017). Ebenfalls bedeutsam sind die Sparkassen, die häufig in kommunaler Trägerschaft und regional verankert für die Kämmereien feste Ansprechpartnerinnen sind.[11] Somit sind ca. drei Viertel der Kommunalfinanzierung durch öffentlich-rechtliche Banken geprägt. Nachgelassen hat die Bedeutung der Realkreditinstitute, deren Hypotheken- und Pfandbriefgeschäft unter Druck geraten ist (Kesseler, 2018) und damit ebenso das Kommunalgeschäft beeinträchtigt hat.[12] Auch die sonstigen Bankengruppen, unter ihnen die großen Geschäftsbanken ebenso wie die Genossenschaftsbanken, spielten in der Kommunalfinanzierung in den vergangenen Jahren nur eine kleinere Rolle. Hier wird das Engagement in der Kommunalfinanzierung stärker durch geschäftsstrategische Entscheidungen bestimmt,

10 Die Zinsbelastung ist von 4,2 Mrd. Euro bei einer Gesamtverschuldung von 113,8 Mrd. Euro im Jahr 2009 auf 2,6 Mrd. Euro bei einem Schuldenstand von 131,4 Mrd. Euro im Jahr 2019 gesunken. Dies entspricht einem Rückgang der Durchschnittsverzinsung von 3,7 % auf 1,9 %. Diese Durchschnittsverzinsung spiegelt nicht die tatsächlichen Kreditkonditionen wider, da Stichtage und Laufzeiten nicht übereinstimmen (Burth, 2017). Eine genauere Aufschlüsselung der Zinskonditionen ist aufgrund fehlender Datenbasis für einzelne Kommunen kaum möglich.

11 Die starke Rolle öffentlich-rechtlicher Institute in der Kommunalfinanzierung wird durchaus kritisch gesehen, insbesondere auch im Hinblick auf die in kommunaler Trägerschaft befindlichen Sparkassen, weil hier eine unzureichende Kontrolle oder Klumpenrisiken vermutet werden (SVR, 2013; oder Deutsche Bundesbank, 2016).

12 Nichtsdestotrotz ist der öffentliche Pfandbrief, dem Kommunaldarlehen als Deckungsmasse zugrunde liegen können, nach wie vor quantitativ bedeutend (Mühlbauer, 2015), der jedoch seit 2005 auch von anderen Bankengruppen ausgereicht wird.

Abbildung 3

**Marktanteile ausgewählter Bankengruppen insgesamt und für Laufzeiten < 1 Jahr**

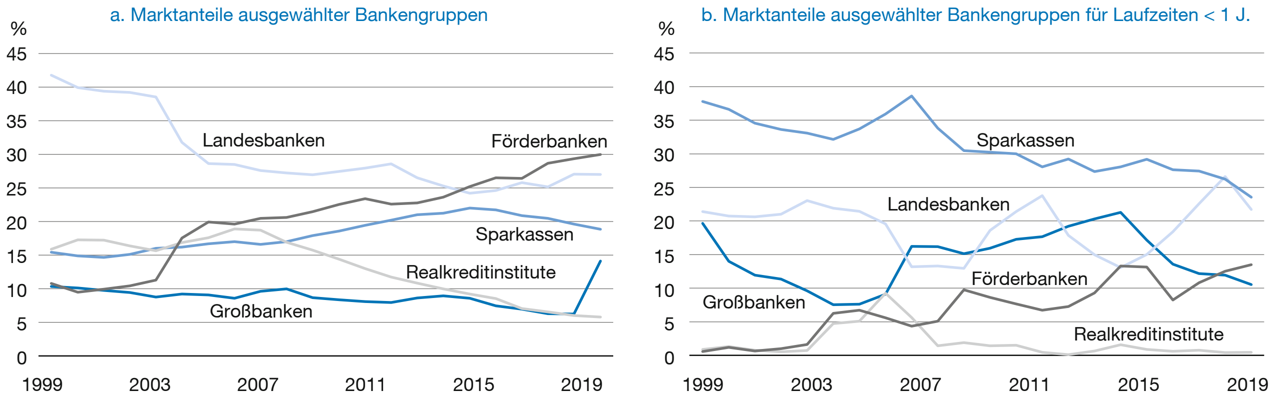

Anmerkungen: Dargestellt ist der Anteil ausgewählter Bankengruppen am Gesamtvolumen der kommunalen Kredite beim nicht-öffentlichen Bereich (Abbildung 3a) bzw. der Kreditverschuldung mit Laufzeiten von bis zu einem Jahr beim nicht-öffentlichen Bereich (Abbildung 3b).

Quelle: eigene Darstellung nach Bankenstatistik (v. J.); Deutsche Bundesbank.

wobei die Kommunalfinanzierung als margenschwaches, aber sicher geltendes Geschäftsgebiet häufig eine Art „Residualfunktion" zu den profitableren, aber risikoreicheren Geschäftsfeldern einnimmt.[13]

Die unterschiedliche geschäftsstrategische Ausrichtung der Bankengruppen zeigt sich auch bei den Anteilen nach Laufzeiten. Während bei den Langfristkrediten vor allem die Förder- und Landesbanken involviert sind, spielen bei den kurzlaufenden Krediten die Sparkassen eine wichtige Rolle. Zuletzt nahm aufgrund der guten konjunkturellen Lage die Höhe dieser Kredite aber ab und die Anteile der Bankengruppen glichen sich zunehmend an (vgl. Abbildung 3b). Ein Großteil der kommunalen Kredite weist jedoch laut Bankenstatistik eine lange Laufzeit von mehr als fünf Jahren auf. Dies ist nicht verwunderlich, weil Kommunen Kredite vor allem für investive Zwecke aufnehmen und in Anbetracht der langen Lebensdauer ihrer Anlagegüter an einer möglichst langlaufenden Infrastrukturfinanzierung interessiert sind. Der Anteil dieser Kredite beträgt zwischen 80 % und 90 %, wobei in der Dekade seit 2009 der Anteil der kurz- und mittelfristigen Kredite auf rund ein Fünftel anstieg, dann aber wieder abnahm.[14]

## Auswirkungen auf die kommunalen Haushalte

Die Corona-Krise beendet die bisher positive Entwicklung kommunaler Haushaltsüberschüsse der letzten Jahre (Brand und Steinbrecher, 2020b). Absehbar sind steigende Ausgaben bei sinkenden Einnahmen, sodass es wahrscheinlicher wird, dass die Haushaltsdefizite durch Konsolidierungsmaßnahmen geschlossen werden müssen. Die Sorge ist, dass unter diesen finanziellen Einschnitten vor allem die Investitionen leiden, weil diese disponibel sind; ein Muster das sich schon in der Finanzkrise gezeigt hat und für viele Jahre nachwirkte. Erste Einschätzungen der Kommunen deuten tatsächlich auf eine Kürzung von Investitionen als Reaktion auf die Krise hin (Brand, Steinbrecher und Krone, 2020).[15] Gleichzeitig wollen die Kommunen über eine verstärkte Kreditfinanzierung reagieren. Für die kommunale Kreditnachfrage sind deshalb zwei gegenläufige Trends maßgeblich, die sich aus

13 Ein wesentliches Risiko für die Profitabilität des Kommunalgeschäfts stellt eine mögliche Verschärfung der Regulatorik dar mit dem Zwang, Kommunalkredite mit (mehr) Eigenkapitel hinterlegen zu müssen (Schlüter, 2016). Bereits Basel III hat den Kommunalkredit unter Druck gesetzt, weil sich dieser nun in stärkerer Konkurrenz zu margenstärkeren Geschäften wiederfindet (Bundesverband Öffentlicher Banken Deutschlands, 2017). Allerdings ist die unterstellte niedrige Profitabilität des Kommunalkredits bis heute unklar, weil anders als in vielen anderen Geschäftsbereichen der Banken keine bedeutenden Risiken im Kommunalgeschäft eingetreten sind. Dem Kommunalgeschäft werden deshalb auch Wachstumschancen zugebilligt (Nitsche, 2018; oder Frien, 2018).

14 Aufgrund der Finanzkrise waren langfristige Kredite schwieriger zu bekommen und die Einführung von Basel III zwang die Banken, sich möglichst viel Handlungsspielraum durch kurze Laufzeiten zu erhalten. Zudem mussten Kommunen Haushaltsengpässe verstärkt über Kassenkredite decken, bevor ab Mitte der 2010er Jahre die konjunkturelle Erholung wieder Haushaltsüberschüsse ermöglichte.

15 Entgegen der frühen Einschätzungen der Kämmereien deuten erste Daten für 2020 auf um 3 % höhere Investitionsausgaben (nominal) hin, damit aber weniger, als noch vor der Krise geplant (Deutscher Städtetag, 2020).

Abbildung 4

**Entwicklung der Zins-Einnahme-Quote und Entwicklung von Gesamtverschuldung zu Gesamteinnahmen**

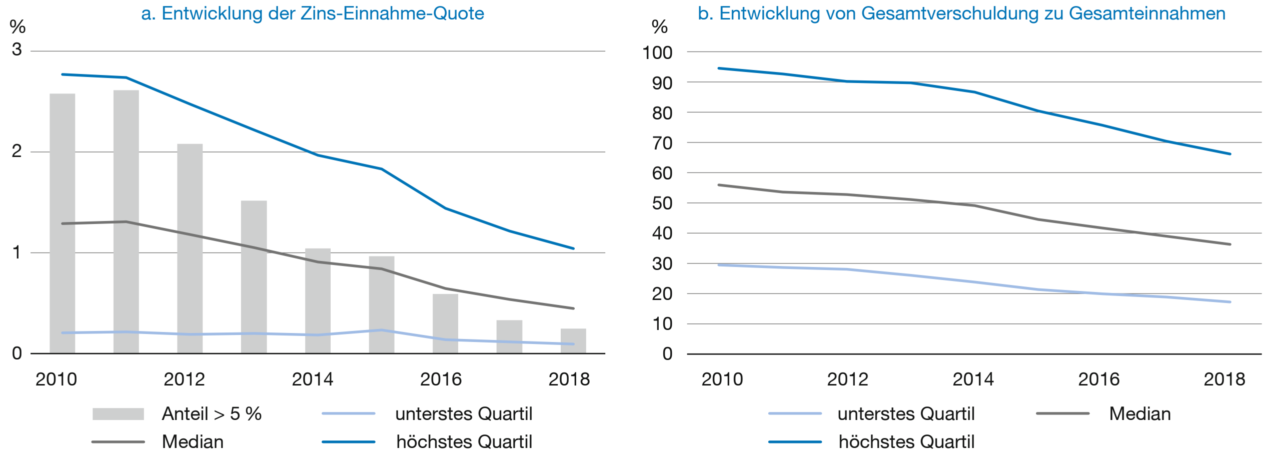

Anmerkungen: Dargestellt ist die Entwicklung Zins-Einnahme-Quote (Zinsausgaben zu Gesamteinnahmen) für alle Gemeinden mit mehr als 2.000 Einwohner:innen anhand des Medians sowie des 25 %- und 75 %-Quartil der Zins-Einnahme-Quote (Abbildung 4a). Darüber hinaus ist in der Abbildung der Anteil der betrachteten Kommunen abgebildet, deren Zins-Einnahme-Quote über 5 % beträgt. In Abbildung 4b ist anhand des Medians sowie des 25 %- und 75 %-Quartil die Entwicklung des Verhältnisses von Verschuldung zu Gesamteinnahmen abgebildet.

Quelle: eigene Darstellung nach Kassen- und Schuldenstatistik (v. J.); Statistisches Bundesamt.

der verschlechterten Haushaltslage ergeben: Einerseits ein steigender Anteil an Krediten an der Finanzierung aufgrund sinkender Überschüsse. Andererseits eine unklare Entwicklung des Investitionsvolumens, das über Kredite kofinanziert wird.

Die Prognosen der Kommunalen Spitzenverbände gehen von erheblichen Defiziten nicht nur im laufenden Jahr, sondern auch in den Folgejahren aus (Greive und Hildebrand, 2020). Bereits die ersten Quartalsdaten zeigen an, dass die Fehlbeträge beträchtlich steigen (Statistisches Bundesamt, 2020c). So fällt das Finanzierungsdefizit mit -9,7 Mrd. Euro im ersten Halbjahr 2020 deutlich schlechter aus als im gleichen Zeitraum des Vorjahres, als es bei -0,2 Mrd. Euro lag, und damit auch schlechter als in den Krisenjahren 2009 und 2010. Ein bedeutender Treiber dafür sind die Einnahmeeinbrüche bei der Gewerbesteuer von in der Summe über 20 %.[16] Allerdings sind die Kommunen hierbei – abhängig z. B. von ihrer lokalen Branchenstruktur – sehr unterschiedlich betroffen. Die Einbrüche bei den Steuereinnahmen treffen tendenziell die finanzstarken Kommunen stärker, die aber zugleich häufiger über Rücklagen verfügen und sich bei einem Wirtschaftsaufschwung auch schneller erholen dürften (Kühl und Scheller, 2020).

Um die Kommunalfinanzen in diesem Jahr zu stabilisieren, wurden finanzielle Hilfen von Bund und Ländern auf den Weg gebracht (Bundesministerium der Finanzen, 2020; oder Geißler, 2020). Zu den wichtigsten Maßnahmen zählen die Erstattung der Gewerbesteuerausfälle von einmalig 11,8 Mrd. Euro sowie die weitere Übernahme von nahezu 75 % der Kosten der Unterkunft mit einem geschätzten Volumen von jährlich 3,4 Mrd. Euro. Diese Maßnahmen dürften vorerst helfen, die Planungsunsicherheit zu vermindern, die sich für die kommunale Investitionstätigkeit mindestens so nachteilig auswirken könnte wie die Finanzierungsdefizite an sich. Sollte die Krise jedoch länger andauern, werden die bisher ergriffenen Maßnahmen kaum ausreichen und die Finanzierungsprobleme dürften auch in Kommunen kräftiger zu Buche schlagen, die bislang noch glimpflich durch die Krise gekommen sind. Kommunale Investitionen könnten in diesem Fall wieder stärker in Mitleidenschaft gezogen werden als es bislang der Fall ist, mit entsprechenden Konsequenzen für die Nachfrage nach Investitionskrediten.

## Auswirkungen auf den kommunalen Kreditmarkt

Auf der Angebotsseite dürfte sich die Krise auch auf den Markt für Kommunalkredite auswirken, wobei das Geschäft vieler deutscher Banken schon vor der Krise unter Druck geraten war (SVR, 2019; oder Deutsche Bundes-

16 Gerade bei der Gewerbesteuer können Unternehmen noch über lange Zeit Korrekturen melden, die zu Nachzahlungen für die Kommunen führen, sodass eine abschließende Bewertung der Corona-Folgen erst in der weiteren Zukunft möglich sein wird. Nicht grundlos ist die volatile Gewerbesteuer als wichtige Finanzierungsquelle der Kommunen immer wieder Gegenstand der Kritik, ohne dass sich einer der vielen Reformvorschläge bislang durchsetzen konnte.

bank, 2020). Sollten jetzt vermehrt Unternehmen insolvent bzw. Kredite notleidend werden, könnte sich die Lage am Bankenmarkt weiter verschärfen. Möglich wäre dann ein Rückgang an (günstigen) Angeboten von Kommunalkrediten, wenn sich Banken aus der Kommunalfinanzierung zurückziehen. Bislang deutet sich eine derartige Entwicklung aber nicht an, sondern eher wird von einer steigenden Bedeutung der sicheren Anlagemöglichkeit im Kommunalbereich berichtet (Geldinstitute, 2020; oder ZfK, 2020). Dies dürfte im Besonderen für die öffentlich-rechtlichen Kreditinstitute gelten, deren Engagement in der Kommunalfinanzierung vor allem politisch definierten Zielen folgt. Hier sind gerade für die Förderbanken zuletzt durch die Politik vermehrt Aufträge formuliert worden, über zusätzliche Förderprogramme die Transformation der Kommunen beispielsweise in Sachen Klimaschutz, Digitalisierung oder Demografie zu unterstützen, sodass zumindest für diese Bankengruppen eher von einer Ausweitung des Kommunalgeschäfts auszugehen ist.

Mit Blick auf die Kreditnehmenden auf der Nachfrageseite wird die Schuldentragfähigkeit nach Corona wahrscheinlich wieder größeres Augenmerk auf sich ziehen. Durch die gute konjunkturelle Lage der Vorjahre ist allerdings ein Verschuldungsspielraum in vielen Kommunen gegeben, sodass keine grundsätzlichen Zweifel an der guten Bonität der deutschen Kommunen angebracht erscheinen. Anhand verschiedener Tragfähigkeitsindikatoren wird deutlich, dass die Kommunen ihre Schuldenlast im Vor-Corona-Zeitraum deutlich reduzieren konnten (vgl. Abbildung 4). Die Fähigkeit, fiskalisch auf die Krise zu reagieren, hat sich in den Jahren 2010 bis 2018 für die meisten Kommunen somit signifikant verbessert. Jedoch zeigt sich trotz gesunkener Zinsen auch weiterhin eine große Streuung bei den Zinslasten zwischen den Kommunen. Als Folge der Corona-Krise wird nicht nur auf kommunaler Ebene, sondern vor allem auch bei Bund und Ländern eine stärkere Konsolidierungsnotwendigkeit deutlich, beispielsweise um die Schuldenbremse wieder einzuhalten und die aufgrund der Krise aufgenommenen Schulden wieder zu tilgen (Hüther und Südekum, 2017). Dies könnte auch die Kommunalhaushalte in Mitleidenschaft ziehen, wenn Bund und Länder ihre Einsparnotwendigkeiten überwälzen, indem sie beispielsweise Zuweisungen an die Kommunen reduzieren.

## Fazit

Der Kommunalkredit ist eine wichtige Säule für die Finanzierung kommunaler Aufgaben. Gerade in der Krise sollte die kommunale Investitionsfähigkeit gestärkt werden, um wirtschaftliche Folgen abzufedern und gleichzeitig wichtige gesellschaftliche Transformationen voranzutreiben (Köhler-Geib, 2020). Corona hat aber regional unterschiedliche, kurz- und langfristige Folgen (Brand, Steinbrecher und Krone, 2020; oder Freier und Geißler, 2020).[17] Auf die regionalen Disparitäten muss durch regional angepasste, zielgruppenspezifische Maßnahmen in der Krise reagiert werden. Noch bleibt abzuwarten, wie groß die Folgen der Corona-Krise tatsächlich ausfallen und inwieweit die Hilfen von Bund und Ländern wirken. Die Frage inwiefern die Investitionsfähigkeit der Kommunen nachhaltig beeinträchtigt wird, kann somit noch nicht abschließend beantwortet werden.

Positiv stimmt in jedem Fall, dass sich die Schuldentragfähigkeit der Kommunen im Vorkrisenzeitraum deutlich verbessert hat. Der Kommunalkredit kann somit auch in den kommenden Jahren eine wichtige Rolle bei der Sicherstellung der Investitionen spielen. Entscheidend ist dafür, die Planungssicherheit für die Investitionen der Kommunen zu erhöhen. Mit Blick auf den gut entwickelten Markt für Kommunalfinanzierung sollte dann auch die Kreditnachfrage stabil bleiben. Insofern wird die Corona-Krise keine Zeitenwende in der Kommunalfinanzierung einläuten, sondern lenkt den Blick auf die strukturellen Risiken und Disparitäten in den Kommunalfinanzen, die bereits vorher existierten und nun wieder deutlicher zu Tage treten.

17 Das Bild wird noch unübersichtlicher, wenn neben Wirtschafts- und Kommunalstrukturen (Kommunalisierungsgrad) auch noch das kommunale Vorfeld in Form der Zweckverbände und Unternehmen (Auslagerungsgrad) berücksichtigt wird (Brand und Steinbrecher, 2019b; oder Boettcher et al., 2019).

## References

[CR1] Albers, H. und R. Rohloff (2007), *Finanzierung kommunaler Investitionen*, Kommunal- und Schul-Verlag Wiesbaden.

[CR2] Bender, C. et al. (2020), Auswirkungen der Corona-Pandemie auf die kommunalen Haushalte und Infrastrukturen, *KOMKIS Report*, Nr. 7.

[CR3] Boettcher, F. et al. (2019), Kommunale Finanzen im Jahr 2018; in Bertelsmann Stiftung (Hrsg.), *Kommunaler Finanzreport 2019*, 10 ff.

[CR4] Brand S (2015). Paradigmenwechsel in der Kommunalfinanzierung — der lange Schatten der Finanzkrise. Wirtschaftsdienst.

[CR5] Brand, S. (2016), Kommunale Kassenkredite — trotz niedriger Zinsen keine Entwarnung, *KfW Research Fokus Volkswirtschaft*, Nr. 114.

[CR6] Brand, S. und J. Steinbrecher (2017a), Paradigmenwechsel in der Kommunalfinanzierung? Aktuelle Entwicklungen beim Kommunalkredit, in M. Junkernheinrich et al. (Hrsg.), *Jahrbuch für öffentliche Finanzen 2017*, 425 ff.

[CR7] Brand, S. und J. Steinbrecher (2017b), Rückgang des Investitionsrückstands — Trendwende oder nur Schönwetterlage?, *KfW Research Fokus Volkswirtschaft*, Nr. 195.

[CR8] Brand, S. und J. Steinbrecher (2018a), Kommunales Altschuldenproblem: Abbau der Kassenkredite ist nur ein Teil der Lösung, *KfW Research Fokus Volkswirtschaft*, Nr. 203.

[CR9] Brand S, Steinbrecher J (2018). Kommunaler Altschuldenfonds: Verringerung der effektiven Schuldenlast anstreben. Wirtschaftsdienst.

[CR10] Brand, S. und J. Steinbrecher (2019a), Green Bonds — nachhaltige Alternative für die kommunale Infrastrukturfinanzierung?, *KfW Research Fokus Volkswirtschaft*, Nr. 245.

[CR11] Brand, S. und J. Steinbrecher (2019b), Kommunale Auslagerungen: ein Spiegelbild regionaler Vielfalt, *KfW Research Fokus Volkswirtschaft*, Nr. 268.

[CR12] Brand, S. und J. Steinbrecher (2020a), Kommunale Investitionen: Preiseffekte am Bau „fressen“ Zinsentlastung auf, *KfW Research Fokus Volkswirtschaft*, Nr. 281.

[CR13] Brand, S. und J. Steinbrecher (2020b), Corona und die Kommunalfinanzen — Plötzlicher Absturz nach einem langsamen Sinkflug, *Immobilien & Finanzierung*, Nr. 10, 463 ff.

[CR14] Brand, S., J. Steinbrecher und E. Krone (2020), Kommunalfinanzen in der Corona-Krise: Einbruch erwartet, Investitionen unter Druck, *KfW Research Fokus Volkswirtschaft*, Nr. 289.

[CR15] Bundesministerium der Finanzen (2020), Die Finanzsituation der Kommunen — gemeinsam aus der Krise, *BMF-Monatsbericht Oktober 2020*.

[CR16] Bundesverband Öffentlicher Banken Deutschlands (2017), *Auswirkung der Leverage Ratio auf die Finanzierung der Kommunen*.

[CR17] Burth, A. (2017), Durchschnittszinssätze der Kommunen im EU-Vergleich, *Weblog Haushaltssteuerung.de* (17. Mai 2017).

[CR18] Deutsche Bundesbank (2016), Gemeindefinanzen: Entwicklung und ausgewählte Aspekte, *Monatsbericht Oktober 2016.*

[CR19] Deutsche Bundesbank (2020), Die Ertragslage der deutschen Kreditinstitute im Jahr 2019, *Monatsbericht September 2020.*

[CR20] Deutscher Städtetag (2020), Finanzen der Kommunen für das laufende Jahr stabilisiert — Blick auf 2021 und 2022 bereitet allerdings große Sorge, *Pressemitteilung*, 3. November.

[CR21] Freier R, Geißler R (2020). Kommunale Finanzen in der Corona-Krise: Effekte und Reaktionen. Wirtschaftsdienst.

[CR22] Freier, R. und V. Grass (2013), Kommunale Verschuldung in Deutschland: Struktur verstehen — Risiken einschätzen, *DIW Wochenbericht*, Nr. 16.

[CR23] Frien, B. (2018), Bankgeschäft mit dem Konzern Kommune. Marktumfeld, Spieler und Herausforderungen, Finance Think Tank (Hrsg.).

[CR24] Geißler, R. (2020), Kommunale Finanzen in der Corona-Krise: Was tun die Länder?, *blog.wegweiser-kommune.de* (2. Juli 2020).

[CR25] Geldinstitute (2020), Kommunalfinanzierung bleibt für Banken ein attraktives Geschäftsfeld, *Meldung*, 8. Mai.

[CR26] Greive, M. und J. Hildebrand (2020), Kommunen fürchten Finanzloch von zehn Milliarden Euro in 2021, *Handelsblatt*, 2. November.

[CR27] Handelsblatt (2017), Anleger stecken ihr Geld in Straßen, *Meldung*, 1. Juni.

[CR28] Hüther M, Südekum J (2020). Die Schuldenbremse nach der Corona-Krise. Wirtschaftsdienst.

[CR29] Keck, J. (2017), Förderbanken — verlässliche und kreative Finanzierungspartner der Kommunen, *Immobilien & Finanzierung*, Nr. 8, 514.

[CR30] Kesseler, B. (2018), Besonderheiten des deutschen Bankenmarktes, die Bedeutung der Landesbanken und die spezifischen Anforderungen der Wirtschaft, zugelassene Dissertation, Otto-von-Guericke-Universität.

[CR31] Köhler-Geib, F. (2020), Wie weiter? Worauf es in der Corona-Krise jetzt ankommt, *KfW Research Positionspapier*, 20. August.

[CR32] Köstler, L. (2020), Immer mehr Banken erwägen Kredite mit Negativzins, *Behörden-Spiegel*, 6. Januar.

[CR33] Krone, E. und H. Scheller (2020), *KfW-Kommunalpanel 2020*, KfW Bankengruppe (Hrsg.).

[CR34] Kühl, C. und H. Scheller (2020), Kommunalfinanzen nach Corona: Ungleichheiten nehmen zu, *Difu Berichte*, Nr. 3.

[CR35] Mühlbauer, F. (2015), Kommunalkredite sind Stabilitätsanker, *BörsenZeitung*, 18. Juli.

[CR36] Nitsche, S. (2018), DNK exklusiv: Banker bauen auf Kommunalgeschäft, *Der Neue Kämmerer*, 7. August.

[CR37] Osman, Y. (2008), Banken lassen Kommunen im Stich, *Handelsblatt*, 26. November.

[CR38] Rehm, H. und S. Matern-Rehm (2010), *Kommunalfinanzen*, VS Verlag für Sozialwissenschaften Wiesbaden.

[CR39] Rehm, H. und M. Tholen (2008), *Kommunalverschuldung — Befund, Probleme, Perspektiven*, Berliner Wissenschafts-Verlag.

[CR40] Scheller, H. und S. Schneider (2017), *KfW-Kommunalpanel 2017*, KfW Bankengruppe (Hrsg.).

[CR41] Schiereck, D. und P. Wittmann (2018), Die Kreditnehmersicht auf plattformgestützte Kommunalkredite, *Immobilien & Finanzierung*, Nr. 10, 45.

[CR42] Schlüter, K. (2016), Basel IV: Kreditengpass durch strengere Regulierung?, *Der Neue Kämmerer*, 3. November.

[CR43] Schwarting, G. (2019), *Der kommunale Haushalt*, Erich Schmidt Verlag Berlin.

[CR44] Seikel, D. (2013), *Der Kampf um öffentlich-rechtliche Banken.*

[CR45] Statistisches Bundesamt (2020a), Vierteljährliche Kassenstatistik 1.-4. Quartal 2019, Fachserie 14, Reihe 2.

[CR46] Statistisches Bundesamt (2020b), Schuldenstand des Öffentlichen Gesamthaushaltes 4. Viertelj. 2019, Fachserie 14, Reihe 5 (Die aktuelleren Quartalszahlen entstammen der Vorläufigen Schuldenstatistik der Reihe 5.2).

[CR47] Statistisches Bundesamt (2020c), Kommunen schließen 1. Halbjahr 2020 mit Finanzierungsdefizit von 9,7 Milliarden Euro ab, *Pressemitteilung*, Nr. 385, 1. Oktober.

[CR48] SVR (Sachverständigenrat zur Begutachtung der gesamtwirtschaftlichen Entwicklung) (2013), Gegen eine rückwärtsgewandte Wirtschaftspolitik, *Jahresgutachten 2013/14*.

[CR49] SVR (Sachverständigenrat zur Begutachtung der gesamtwirtschaftlichen Entwicklung) (2019), Banken vor zyklischen und strukturellen Herausforderungen, *Jahresgutachten 2019/2020.*

[CR50] ZfK (Zeitung für kommunale Wirtschaft) (2020), Corona-Krise: Wird die Kommunalfinanzierung jetzt digital?, *Meldung*, 23. April.

[CR51] Zimmermann, H. (2006), Kommunale Verschuldung — wozu?, Wirtschaftsdienst, 86(6), 391 ff.

[CR52] Zimmermann, H. und T. Döring (2019), *Kommunalfinanzen*, Berliner Wissenschafts-Verlag.

